# A Drug Delivery to Improve Prognosis of Traumatic Brain Injury Mice Through Mouse‐Derived Nerve Growth Factor Coated by a Nanoparticle

**DOI:** 10.1111/cns.70603

**Published:** 2025-10-02

**Authors:** Ruichen Zhao, Shiying Dong, Di Wu, Yu Tian, Jiangyuan Yuan, Liang Mi, Chenrui Wu, Shiao Tong, Rongcai Jiang

**Affiliations:** ^1^ Department of Neurosurgery Tianjin Medical University General Hospital Tianjin China; ^2^ Department of Neurosurgery, Xuanwu Hospital Capital Medical University Beijing China

**Keywords:** blood–brain barrier, glymphatic system, nerve growth factor, traumatic brain injury

## Abstract

**Objective:**

The large molecular weight and limited permeability of mouse‐derived nerve growth factor (mNGF) across the blood–brain barrier (BBB) have restricted its therapeutic use after brain injury. We therefore hypothesized that encapsulating mNGF in nanoparticles would facilitate BBB transit, increase delivery to the brain parenchyma, and consequently improve the treatment of traumatic brain injury (TBI).

**Methods:**

Nanoparticles were used to encapsulate the high‐molecular‐weight protein mNGF to improve its delivery. Traumatic brain injury (TBI) was induced in mice, which were then allocated to four groups, including a sham group. Intramuscular injections of mNGF—either free or nanoparticle‐encapsulated—were administered. To elucidate the mechanism of action, the aquaporin‐4 inhibitor 2‐nicotinamide‐1,3,4‐thiadiazole (TGN‐020) was additionally given to the nanoparticle group. Glymphatic function (cerebrospinal fluid influx and efflux) was quantified by immunofluorescence. Blood–brain barrier integrity, peri‐lesional parenchymal structure, and axonal repair were examined using Evans blue extravasation, immunofluorescence, and Western blotting. Neuronal apoptosis and focal neurological damage were measured with TUNEL staining and Western blot analysis. Functional outcomes were assessed with the modified Neurological Severity Score, rotarod performance, and the Morris water maze.

**Results:**

Nanoparticle encapsulation markedly increased the amount of mNGF that reached the brain parenchyma relative to conventional administration. Enhanced delivery enabled substantially more exogenous mNGF to traverse the BBB in TBI mice than did uncoated mNGF. The treatment attenuated TBI‐induced neuronal apoptosis, up‐regulated genes involved in neurogenesis and myelinogenesis, restored glymphatic inflow and outflow, repaired BBB structure and function, and mitigated cognitive deficits. These benefits were abolished by the aquaporin‐4 inhibitor TGN‐020, indicating that mNGF improves TBI outcome by correcting AQP4 dysfunction. To our knowledge, this is the first demonstration that nanocrystallized mNGF can cross the BBB efficiently after TBI and thereby foster neural repair and functional recovery.

## Graphical abstract

This study is the first to demonstrate that mNGF, when nanocrystallized, crosses the BBB more effectively in TBI mice, subsequently aiding the repair and functional recovery of the nervous system post‐injury.

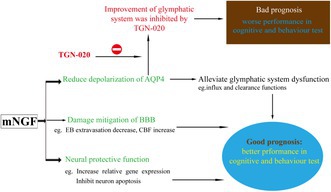

## 1 Introduction

Traumatic brain injury (TBI) is most often caused by road traffic accidents, sports trauma, combat violence, and accidental falls. These insults precipitate extensive neuronal loss, pronounced neuroinflammation, and subsequent cognitive decline [1]. During the reparative phase, glial fibrillary acidic protein (GFAP)‐rich scar tissue disrupts the perivascular polarization of aquaporin‐4 (AQP4), undermining the glymphatic pathway that clears metabolic waste from the central nervous system. Impaired clearance permits the accumulation of neurotoxic species such as amyloid‐β and tau, thereby promoting secondary neuropathology [2]. Despite pharmacological advances, no universally accepted therapy for TBI exists. Consequently, restoring glymphatic function with nanoscale nerve growth factor (NGF) has been proposed as a novel therapeutic approach.

The glymphatic system, responsible for clearing metabolic waste, is vital for protecting the central nervous system (CNS) from toxic accumulation [3]. Aquaporin‐4 (AQP4), concentrated in the astrocytic end‐feet that line perivascular spaces, mediates fluid exchange between cerebrospinal fluid (CSF) and interstitial fluid (ISF) [4]. Loss of AQP4 polarization impairs this clearance pathway, allowing neurotoxic species such as amyloid‐β and tau to accumulate. Growing evidence links AQP4 depolarization‐driven waste elimination failure to neurodegenerative and neuroinflammatory disorders, including Alzheimer's disease, Parkinson's disease, neuromyelitis optica, and TBI [5, 6, 7].

NGF, a pivotal neurotrophic molecule, has been administered directly in clinical trials for TBI treatment [8]. NGF drives astrocytes toward a neuroprotective phenotype, thereby limiting the detrimental effects of excessive glial scarring [9]. It also acts as a pro‐angiogenic agent, up‐regulating vascular endothelial growth factor, which supports endothelial proliferation and migration; the resulting relief of hypoxia enhances waste clearance and improves prognosis in stressed rats [10]. However, the blood–brain barrier severely restricts the delivery of this large molecule, casting doubt on its reparative efficacy in the CNS [11]. This limitation—together with the scarcity of randomized clinical trials—has hindered the clinical translation of NGF. An efficient delivery strategy is therefore still needed, and recent advances in nanotechnology suggest that nanoscale carriers can traverse the blood–brain barrier (BBB) and convey therapeutics to target tissues.

N‐Butyl cyanoacrylate (NBCA), widely used as a surgical tissue adhesive, has also been investigated as a precursor for nanoparticle synthesis [12]. Its polymer, polybutyl cyanoacrylate (PBCA), can ferry large molecules across the BBB into the brain parenchyma in both in vivo and in vitro settings [13, 14]. PBCA nanoparticles are inexpensive, easy to manufacture, and have shown consistent safety and efficacy for decades, making them an attractive drug‐delivery platform. In this study, we employ PBCA to encapsulate mNGF and test whether the resulting formulation can traverse the BBB and lessen acute damage in a severe TBI model.

## 2 Methods and Materials

### 2.1 Animals

The ethics approval statement was labeled as No. IRB2025‐DW‐41. All procedures were approved by the Animal Care and Use Committee of Tianjin Medical University General Hospital and complied with the NIH Guide for the Care and Use of Laboratory Animals. Male C57BL/6 mice (HFK Bioscience Corporation) aged 8 to 12 weeks and weighing 20–25 g were housed under controlled conditions (temperature 18°C–22°C, relative humidity 50%–60%) with a 12‐h light/dark cycle and free access to food and water.

### 2.2 Preparation for PBCA Nanoparticles

Nanocarriers were synthesized as described previously [15]. Briefly, 1 mL of NBCA glue (Shanghai Dowtech Pharma Science & Technology Co., China) was added to 100 mL of dextran 70,000 (0.5% w/v) adjusted to pH 2.5 with HCl, and the mixture was magnetically stirred at room temperature for 8 h to promote polymerization. The cloudy suspension was neutralized with 1 N NaOH and filtered through 0.8 μm paper to remove debris; the filtrate (“raw liquid”) was centrifuged at 16 *g* for 30 min to collect PBCA nanoparticles. Pellets were washed by sonication in deionized water (10 min), air‐dried at room temperature, weighed, and redispersed in deionized water to yield a 0.2 mg/mL PBCA suspension (“centrifuged liquid”) [16]. Particle size distribution was determined with a Zetasizer (FEI Tecnai F20, USA).

### 2.3 Production of Polysorbate 80‐Coated mNGF‐Loaded PBCA Nanoparticles

Commercial mNGF (NOBEX, SINOBIOWAY BIOMEDICINE CO.) was labeled with fluorescein isothiocyanate (FITC, green) to track the protein after delivery. Conjugation was performed in a mildly alkaline buffer, protected from light, for 4 h. Unbound FITC was removed by dialysis, followed by desalting to obtain FITC‐labeled mNGF. To load mNGF onto PBCA nanoparticles, 10 μg of purified mNGF was added to 1 mL of a 0.2 mg/mL PBCA suspension in normal saline, yielding a final mNGF concentration of 10 μg/mL. The mixture was stirred at room temperature for 15 min to promote adsorption to the nanoparticle surface. Prior studies indicate that polysorbate 80 enhances the CNS targeting of PBCA carriers [17]; although the mechanism is not fully understood, apolipoprotein E (ApoE) appears to mediate selective uptake [18]. To exploit this effect, 100 μL of 10% polysorbate 80 was added, giving a final concentration of 1% (w/v). The suspension was stirred for an additional 3 h to ensure complete coating, producing polysorbate‐80‐coated PBCA‐mNGF nanoparticles.

### 2.4 Drug Loading Capacity (DL%) and Encapsulation Efficiency (EE%) Assessment

Encapsulation efficiency was determined by quantifying protein content. High‐performance liquid chromatography (HPLC) is widely used in plasma proteomics and plays a key role in diagnostics [19]. In this study, 1 mL of the turbid PBCA‐mNGF suspension was centrifuged under standard conditions. After removing the supernatant, the pellet was washed three times with saline and air‐dried. To recover non‐encapsulated mNGF, the dried nanoparticles were resuspended in saline; excess Tween‐80 was added to disrupt the nanoparticle shell and release bound mNGF. After a second centrifugation, 10 μL of supernatant was analyzed for free mNGF using an Agilent 1260 HPLC system. Gradient elution was performed at 260 nm with a mobile phase of 70% acetonitrile and 30% 0.1% phosphoric acid at a flow rate of 1 mL/min. A standard curve constructed from five external standards (2, 4, 6, 8, 10 mg/L) showed a linear relationship between peak area and mNGF concentration. mNGF content was calculated from this curve; DL % was defined as the weight ratio of mNGF to nanoparticles, and EE % as the weight ratio of mNGF in the supernatant relative to the total mNGF used in nanoparticle synthesis.

### 2.5 Experiment Design and Drug Administration

**FIGURE 1:**
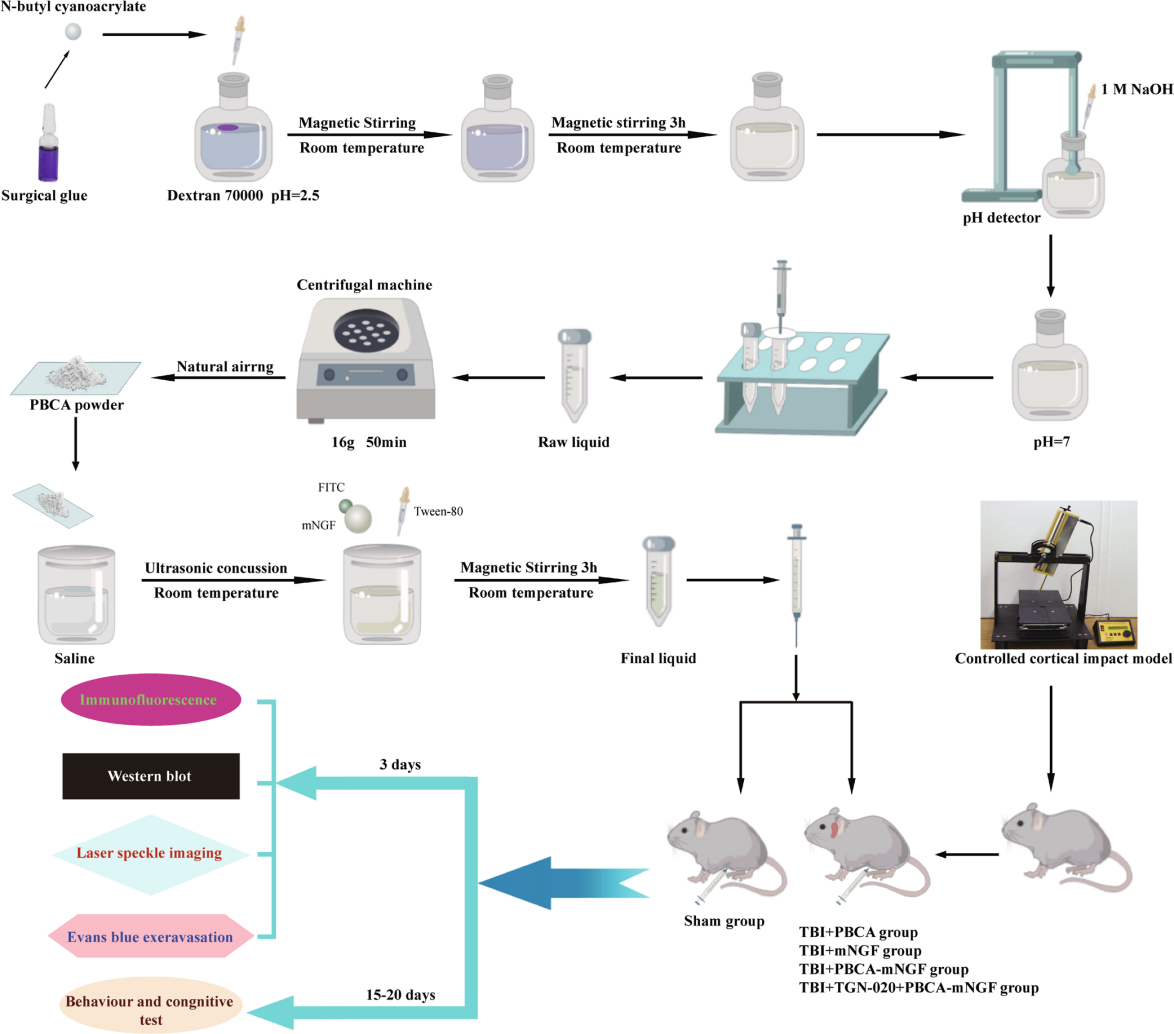
NBCA monomer was extracted from surgical glue by liquid‐phase separation. The solution was neutralized to pH 7, yielding a turbid white suspension that was filtered through 0.8 μm paper to remove impurities; this filtrate was designated “Raw liquid” and stored at 4°C. After ultracentrifugation (16 *g*, 50 min), the supernatant was discarded, and the pellet was washed three times with 1 mL saline under the same conditions. The resulting sediment was air‐dried at room temperature. To obtain a 10 μg/mL preparation, 10 μL purified mNGF was added to 1 mL of the centrifuged liquid. Tween‐80 (1% w/v) was then introduced as an activator to enhance mNGF adsorption, producing the “Activated liquid.” Following 3 h of stirring, the “Final liquid” was prepared and used immediately to preserve mNGF bioactivity. Traumatic brain injury (TBI) was induced with a controlled cortical impact (CCI) device. One hour later, mice in the “TBI + PBCA‐mNGF” group received a 100 μL intramuscular injection of the Final liquid, mirroring clinical Nobex administration. The “TBI + PBCA” group received an equal volume of vehicle, whereas the “TBI + mNGF” group received 100 μL mNGF solution to match the dose used in the TBI + PBCA‐mNGF group. As a negative control, TGN‐020 was co‐injected with PBCA‐mNGF. Each formulation was administered once. Mice were euthanized three days later for further analyses, and motor as well as cognitive functions were evaluated on day 15 post‐TBI as previously described.

To evaluate the efficacy of PBCA‐mNGF, we used a controlled cortical impact (CCI) model of TBI in mice. Four groups were established—Sham, TBI + PBCA, TBI + mNGF, and TBI + PBCA‐mNGF. Ten mice per group were reserved for behavioral testing, and an additional six per group were used for histologic and biochemical analyses. TBI was induced with a digitally controlled impactor fitted with a 4‐mm flat tip (depth 2.2 mm, velocity 5 m/s, dwell time 200 ms) [20]. An overview of the protocol is provided in Figure 1. Drugs were delivered intramuscularly, consistent with clinical use. For the TBI + mNGF group, 10 μg of purified mNGF was dissolved in 1 mL of saline (10 μg/mL). Because unloaded PBCA could confound outcomes, an empty PBCA suspension identical to the carrier in the TBI + PBCA‐mNGF group (but lacking mNGF) was administered to the TBI + PBCA controls. Each mouse received 100 μL of its assigned formulation. Given the acute nature of TBI and limited data on mNGF therapy, injections were performed 1 h after injury, a time point used in previous studies [5]. mNGF was FITC‐labeled to monitor brain distribution; fluorescence studies show maximal accumulation 4 h after injection, so mice slated for biodistribution analysis were euthanized at that time. Parallel subgroups were created to confirm BBB transit under both injured and uninjured conditions: Sham + mNGF, Sham + PBCA‐mNGF, TBI + mNGF, and TBI + PBCA‐mNGF. Assessments included glymphatic flow, BBB integrity, neuronal survival, and functional recovery. To probe the mechanism, whereby mNGF influences glymphatic drainage, we added a fifth cohort (TBI + TGN‐020 + PBCA‐mNGF). Mice received a single intraperitoneal dose of the AQP4 inhibitor TGN‐020 (200 mg/kg, HY‐W008574; MedChemExpress) concurrently with PBCA‐mNGF, following a published regimen [21]. All treatments were given once during the study. We hypothesized that TGN‐020 would negate the drainage benefits conferred by mNGF, thereby worsening behavioral and cognitive outcomes relative to the TBI + PBCA‐mNGF group.

### 2.6 Hematoxylin–Eosin (HE) Staining

HE staining was performed as described previously [22]. Briefly, mouse organs (heart, liver, spleen, lung, and kidney) were fixed in 4% paraformaldehyde, embedded in paraffin, and sectioned at 8 μm. Sections were stained with an HE kit (G1120; Solarbio) and examined under a light microscope (IX73, Olympus). Each group contained three mice.

### 2.7 Drug Concentration Quantification

To verify drug levels in the CNS, optical density (OD) values in brain homogenates were measured with a microplate reader (Thermo Fisher Scientific, USA). Brain samples were collected as previously described [22]. A standard curve was generated from a series of known concentrations; OD readings from each sample were then converted to mNGF concentrations. Drug levels in the brains of the TBI + mNGF and TBI + PBCA‐mNGF groups (*n* = 3 per group) were calculated, and each measurement was performed in triplicate.

### 2.8 Primary BMEC Permeability Assay

To assess PBCA transport capacity, primary brain microvascular endothelial cells (BMECs) were cultured in Dulbecco's Modified Eagle Medium (DMEM) supplemented with 10% fetal bovine serum at 37°C in 5% CO_2_ to establish a monoculture BBB model [23, 24]. Cells were seeded onto Transwell filters (LABSELECT 14211; 12 mm, 0.4 μm polycarbonate membrane) and grown to confluence. After 2 h of serum‐free starvation, the upper chamber received PBCA‐mNGF (diluted in DMEM; “PBCA‐mNGF” group) or an equivalent concentration of uncoated mNGF (“mNGF” group). Following 4 h of transcellular diffusion, FITC fluorescence in both chambers was measured with the same microplate reader. Relative permeability was calculated as previously reported [25].

### 2.9 In Vivo Tissue‐Targeting Distribution Analysis

In vivo biodistribution was evaluated as described earlier [26]. To determine whether PBCA‐mNGF accumulated at TBI lesions, Cy5.5‐labeled mNGF at the same concentration was encapsulated in PBCA. Intramuscular injection was chosen to avoid variability from different administration routes. Whole‐body imaging was performed with an IVIS Lumina II system (PerkinElmer, USA) at 6, 12, and 24 h post‐injection. Radiant efficiency was quantified with Living Image software version 3.1 (Caliper Life Sciences).

### 2.10 Serum Stability

Although PBCA's physicochemical stability is well documented [27], its biological stability must also be verified. Following a published protocol [28], we evaluated serum stability using the same nanoparticle concentration described in Section 2.2. The suspension medium was replaced with 10% bovine serum albumin (BSA), and samples were stored at room temperature (RT). Particle size was measured on days 0–7 with the identical Zetasizer method (*n* = 3). Statistical procedures are detailed in Section 2.25.

### 2.11 Drug Release

Drug release was assessed as previously described [22]. PBCA‐mNGF prepared in Section 2.3 was placed in dialysis tubing (Slide‐A‐Lyzer, 20 kDa MWCO; Thermo Fisher), a cutoff large enough to permit PBCA‐mNGF passage. The dialysis bag containing 1 mL of formulation was immersed in 100 mL of PBS (pH 7.4) at 37°C. At predetermined intervals (0, 0.25, 0.5, 1, 2, 4, 6, 12, and 24 h), 200 μL of dialysate was withdrawn and replaced with fresh PBS to maintain constant volume. Samples were stored at 4°C until analysis. mNGF concentration was quantified as described in Section 2.4, and cumulative release was plotted with GraphPad Prism 9.0.

### 2.12 Cell Counting Kit‐8 (CCK‐8)

A CCK‐8 assay [29] was used to determine the cytotoxicity of PBCA and free mNGF toward neural cells. Highly differentiated PC12 cells were seeded in 96‐well plates and incubated overnight. PBCA or mNGF was added at sample‐to‐medium ratios ranging from 1:5 to 1:1000. After 24 h of exposure, CCK‐8 reagent (Beyotime, Shanghai, China) was applied for 30 min at 37°C. Optical density was recorded at 450 nm, and cell viability was calculated with GraphPad Prism 9.0.

### 2.13 Blood Biochemical Examination

Blood chemistry was performed as reported previously [30]. On day 7 post‐TBI, whole blood was collected for analysis of WBC, RBC, PLT, HGB, ALT, AST, Cr, and BUN in mice treated with unloaded PBCA or PBCA‐mNGF (*n* = 6 per group). To further evaluate safety, a “3× unloaded PBCA” solution (threefold the carrier concentration) was administered as an overdose control [31].

### 2.14 Intracisternal Tracer Infusions

Lymphatic function was assessed by infusing a fluorescent tracer into the cerebrospinal fluid (CSF), as described previously [13]. Mice were anesthetized and secured in a stereotaxic frame. Fluorescent dextran was delivered into the cisterna magna at 1 μL/min for 10 min via a micro‐injection pump. After 30 min of CSF circulation, mice were transcardially perfused with 4% paraformaldehyde. Brains were post‐fixed overnight at 4°C, cryoprotected in 15% and 30% sucrose (24 h each), embedded in optimal cutting temperature compound (Sakura Finetek, USA), and sectioned coronally at 100 μm using a CM1950 cryostat (Leica Biosystems, Germany). To map tracer influx, seven rostro‐caudal levels relative to bregma were analyzed: +1.93, +0.85, −0.47, −1.23, −1.79, −2.45, and −3.07 mm (negative values indicate caudal positions). Fluorescence was imaged with a conventional microscope and quantified in ImageJ [5].

### 2.15 Intraparenchymal Injections

Parenchymal efflux capacity was evaluated as reported previously [32]. A 500‐nL CSF tracer bolus was injected over 10 min at a depth of 2.0, 1.5 lateral, and 2.0 mm posterior to bregma. One hour later, mice were perfused, and sections at −2.06 mm from bregma were processed as in Section 2.14 to quantify tracer clearance.

### 2.16 AQP4 Polarization Evaluation

AQP4 polarization was quantified by measuring immunofluorescence (IF) intensity in perivascular regions. Thresholding defined the percentage of vascular profiles whose AQP4 signal met or exceeded baseline (“AQP4 area %”). Depolarized regions were visualized, and polarization was calculated as:

Polarization%=100−Depolarization%

### 2.17 Laser Speckle Imaging

Cerebral blood flow (CBF) was measured 3 days after TBI with the PeriCam PSI system (Perimed AB, Sweden) following published methods [33]. Under anesthesia, a midline scalp incision exposed the skull. CBF maps were acquired and processed in PIMsoft 1.2. Regions of interest were matched across animals; baseline values were obtained from the Sham group.

### 2.18 EB Extravasation

Blood–brain barrier integrity was evaluated using EB dye [34]. Mice received 2% EB (4 mL/kg, i.v.; Sigma‐Aldrich, E2129) and were euthanized 2 h later. Brains were harvested, homogenized, centrifuged, and the supernatant's absorbance was measured with a spectrofluorophotometer to quantify EB content.

### 2.19 IF

IF staining followed standard protocols [35]. Sections were incubated with primary antibodies—AQP4 (1:500), GFAP (1:500), CD31 (1:500), NeuN (1:500), NF‐200 (1:500), PSD‐95 (1:500), MBP (1:500), Claudin‐5 (1:500), Iba‐1 (1:500), and CD68 (1:500)—stored at −20°C. After washing, species‐specific fluorescent secondary antibodies (stored at 4°C) were applied. Images were captured with a fluorescence microscope and analyzed in ImageJ.

### 2.20 Western Blot (WB)

WB was performed as previously described. Target proteins included zonula occludens‐1 (ZO‐1, 220 kDa), occludin (59 kDa), neuronal nuclei (NeuN, 53 kDa), postsynaptic density‐95 (PSD‐95, 95 kDa), myelin basic protein (MBP, 20 kDa), neurofilament‐200 (NF‐200, 200 kDa), B‐cell lymphoma‐2 (Bcl‐2, 22 kDa), and β‐actin (43 kDa). Lesion‐site lysates were collected, and Sham samples served as controls. After SDS‐PAGE and transfer, polyvinylidene difluoride (PVDF; Millipore) membranes were blocked with 5% skim milk and incubated overnight at 4°C with primary antibodies. HRP‐conjugated secondary antibodies were applied for 1 h, blots were visualized on a ChemiDoc Touch system, and band density was quantified with ImageJ.

### 2.21 TUNEL Staining

Apoptotic neurons were detected with the CF488 TUNEL Cell Apoptosis Detection Kit per the manufacturer's instructions. NeuN immunofluorescence, using the same primary antibody as in WB, identified neurons. The percentage of NeuN‐positive cells that were also TUNEL‐positive was determined with a fluorescence microscope and ImageJ.

### 2.22 Rotarod Test

Motor coordination and balance were assessed on a rotarod (YLS‐4C, Beijing) 1, 3, 5, 7, and 14 days after TBI, following published procedures [36]. Mice were pre‐trained at 5 to 10 rpm for 300 s. During each test, the rod accelerated from 5 to 40 rpm over 300 s; three trials were performed, and the mean latency to fall was recorded.

### 2.23 Morris Water Maze (MWM)

Spatial learning and memory were evaluated with the MWM [37]. Ten mice per group were tested over 14 days. From days 15 to 19, animals were trained to locate a hidden platform; failures after 90 s were guided to the platform. On day 20, the platform was removed, and mice were placed in the opposite quadrant. Tracking with EthoVision XT 13 (Noldus, Netherlands) yielded latency to platform location, platform crossings, dwell time, swim speed, and path efficiency, displayed as heatmaps and trajectories.

### 2.24 Modified Neurological Severity Score (mNSS)

Neurological deficits were scored on days 1, 3, 5, 7, and 14 after injury using the mNSS [35]. Higher scores indicate greater impairment.

### 2.25 Statistical Analysis

Animals were randomly assigned, and all experiments were repeated at least twice by investigators blinded to group allocation. Data are presented as mean ± standard deviation (SD). Normality was checked with the Shapiro–Wilk test. Two‐group comparisons used unpaired, two‐tailed Student's *t*‐tests; multi‐group data were analyzed by one‐ or two‐way ANOVA with Tukey's post hoc test. Significance was set at *p* < 0.05: * < 0.05, ** < 0.01, *** < 0.001, **** < 0.0001.

## 3 Results

### 3.1 Basic Properties of PBCA Nanoparticles

**FIGURE 2:**
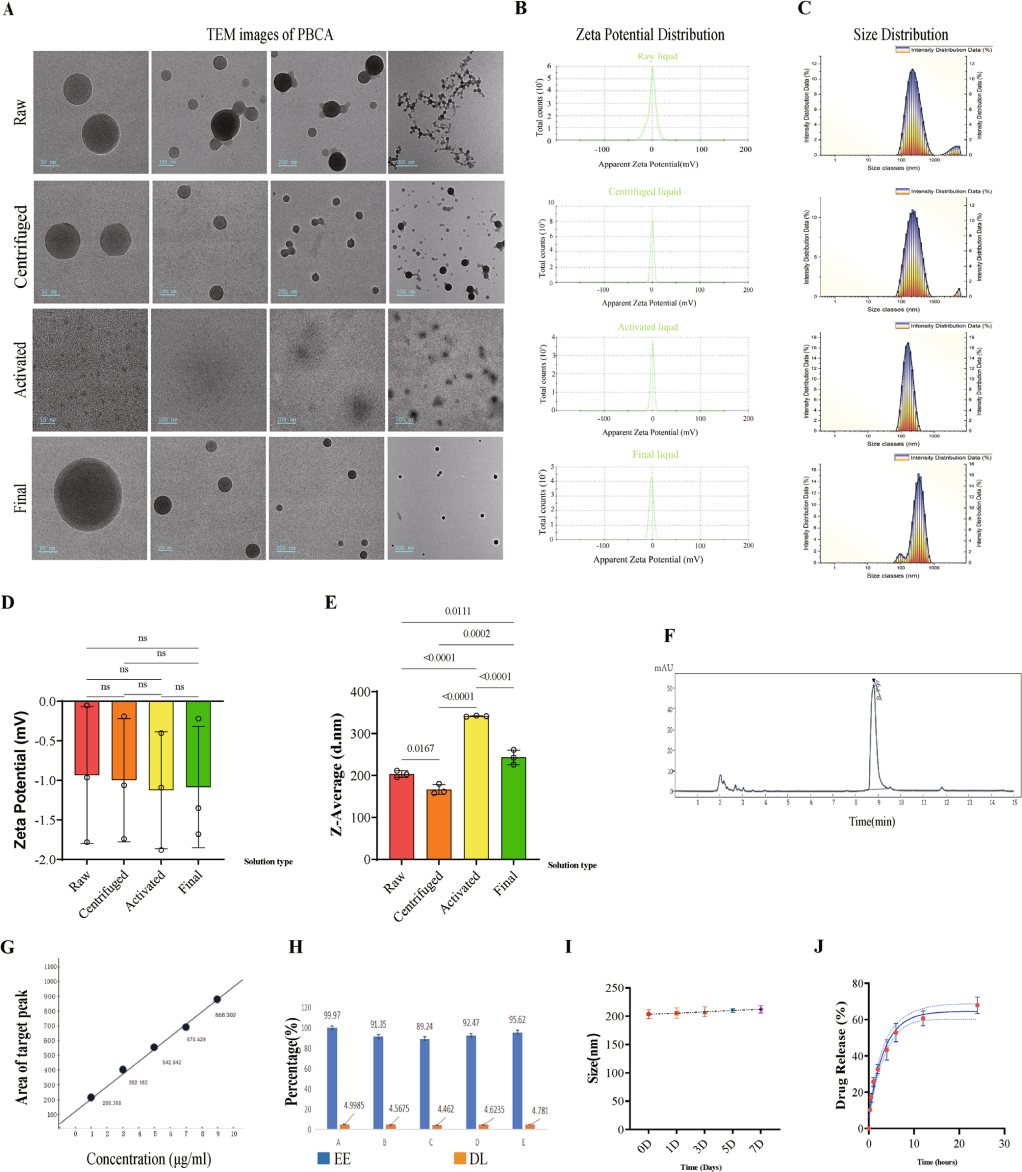
Basic properties of PBCA nanoparticles. (A) TEM images of PBCA particles at four key time points, shown at 50, 100, 200, and 500 nm magnifications. (B) Representative zeta‐potential curves corresponding to panel A. (C) Representative particle‐size distribution curves at the same four time points. (D) Quantification of PBCA zeta potential across the four stages. (E) Quantification of mean particle size across the four stages. (F) Representative HPLC chromatogram of mNGF, with retention time indicated near the peak. (G) Standard curve relating peak area to concentration, generated from five external standards. (H) Encapsulation efficiency (EE %) and drug‐loading capacity (DL %) for PBCA‐mNGF (*n* = 5 “PBCA + mNGF” samples). (I) Serum stability of PBCA over one week (*n* = 3 “Final fluid” samples). (J) In vitro release profile of PBCA‐mNGF (*n* = 3 “Final fluid” samples).

Figure 1 outlines nanoparticle production and administration. Nanoparticle morphology was examined at multiple magnifications with a transmission electron microscope (TEM). PBCA particles in the “Centrifuged” fraction showed less adhesion than those in the “Raw” fraction, yet the mean diameter in both remained 70–100 nm (Figure 2A,Ca,Cb). Ultracentrifugation did not alter zeta potential (Figure 2Ba,b). After Tween‐80 activation, particles appeared more loosely packed (Figure 2A). During magnetic stirring, mNGF adsorbed onto the particles and became cargo.

Zeta potential reflects surface charge; higher absolute values usually confer greater colloidal stability. Here, PBCA potentials ranged from 0 to −1.2 mV, remaining within ±10 mV (Figure 2D), a range consistent with rapid polymerization. Despite Tween‐80 treatment, the zeta potential stayed low, suggesting the particles could readily regain a spherical morphology suitable for drug delivery (Figure 2Bc). Stirring continued for 4 h, and the resulting “Final” suspension was re‐evaluated for intramuscular use. These particles were non‐adhesive and uniformly spherical (Figure 2A). Because Tween‐80 coats the surface, final particles were larger than those in the earlier fractions (Figure 2A,Ba,b,d) yet smaller than those in the “Activated” state, reflecting shape recovery during extended stirring (Figure 2A,Bc,d). With a final zeta potential still within ±10 mV and PBCA degradable to non‐toxic metabolites, particle morphology should remain stable in vivo, facilitating mNGF release and neuroprotection.

HPLC assessed nanocrystallization efficiency and quantified mNGF loading in 1 mL PBCA‐mNGF samples. Five replicates and five external standards generated the calibration curve shown in Figure 2F; the equation Y = 84.65965 X + 23.68560 demonstrated excellent linearity between peak area (Y) and mNGF concentration (X, μg/mL) (Figure 2G). The mean peak area (817.20) corresponded to 9.37 μg/mL loaded mNGF. Encapsulation efficiency (EE %) averaged 93.73%, whereas drug‐loading capacity (DL%) was 4.67%, indicating many empty carriers (Figure 2H). To offset the potential effects of unloaded particles, TBI mice received an equal dose of empty PBCA.

Intrinsic nanoparticle behavior was further characterized (Figure 2J,I). Particle size remained stable for 7 days in 10% serum. In vitro, roughly 50% of mNGF was released within 6 h; after 12 h the rate slowed, and about 65% had been released by 24 h. In vivo release kinetics will be explored in subsequent experiments.

### 3.2 PBCA‐mNGF Had High Biosecurity and an Enhanced Ability to Traverse the BBB and Deliver to Cerebral Parenchyma in TBI Mice

**FIGURE 3:**
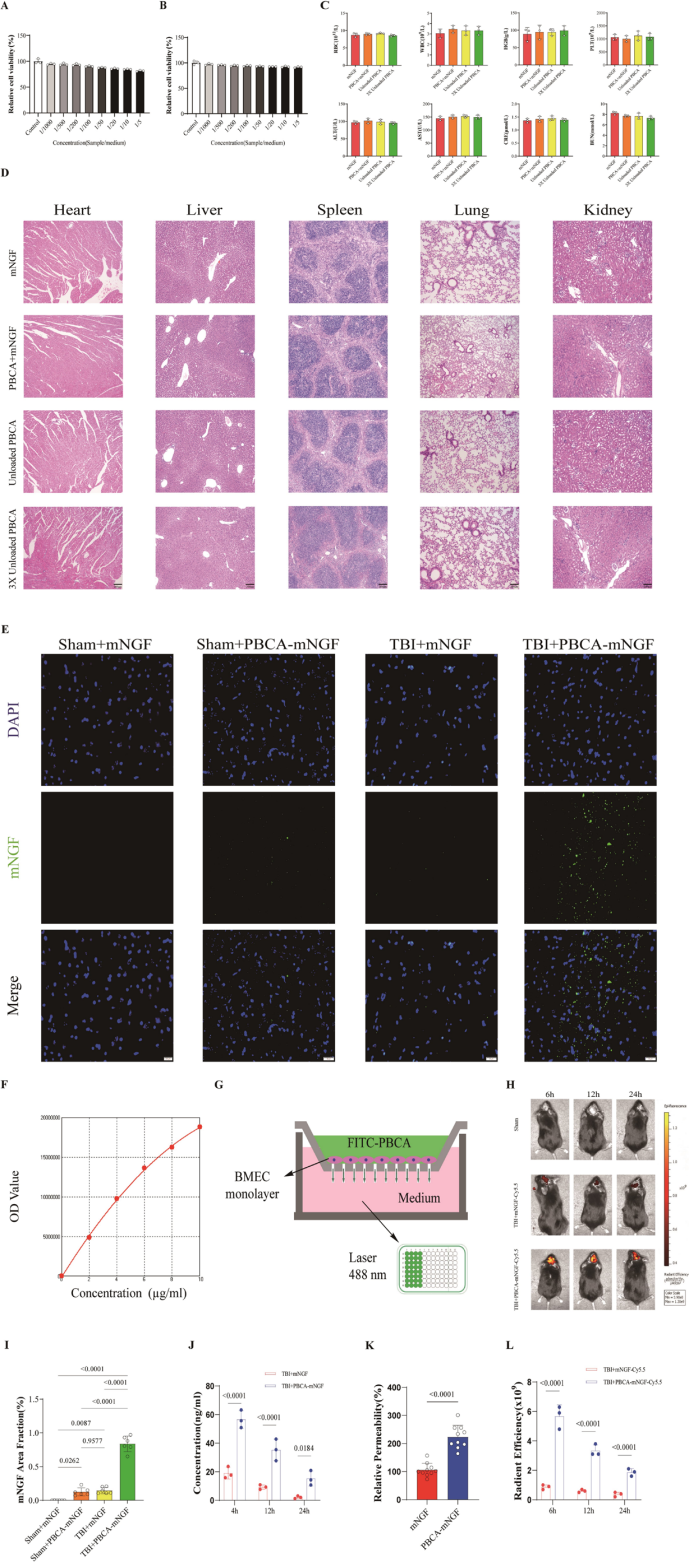
mNGF Exhibits an Enhanced Ability to Traverse the BBB and Reach the Cerebral Parenchyma. (A) Cell‐viability assay for unloaded PBCA (*n* = 3 PBCA samples). (B) Cell‐viability assay for FITC‐labeled mNGF (*n* = 3 mNGF samples). (C) Blood‐biochemistry profiles of mice treated with unloaded PBCA or PBCA‐mNGF on day 7 post‐TBI (*n* = 3 mice per group). (D) HE staining of heart, liver, spleen, lung, and kidney from the same treatment groups (*n* = 3 mice per group). (E) Fluorescence images showing cortical mNGF distribution in the four experimental groups. (F) Standard curve for FITC‐labeled mNGF concentrations in the CNS. (G) Schematic of the primary brain microvascular endothelial cell (BMEC) permeability assay (*n* = 3 mice per group). (H) In vivo imaging of PBCA‐mNGF tissue distribution at 6, 12, and 24 h post‐injection (*n* = 3 mice per time point). (I) Quantification of the FITC‐labeled mNGF‐positive area in brain sections from panel E (*n* = 6 mice per group). (J) Drug concentrations in TBI brains at 4 h, 12 h, and 24 h post‐injection. (K) Permeability of cultured BMEC monolayers to free mNGF versus PBCA‐mNGF (*n* = 10 replicates per group). (L) Quantitative analysis of PBCA‐mNGF fluorescence in mouse brains from the in vivo imaging study.

We assessed the toxicity of PBCA and mNGF both in vivo and in vitro. For neuronal evaluation, PC12 cells underwent a cytotoxicity assay. The data indicated that neither unloaded PBCA nor FITC‐labeled mNGF significantly affected cell viability (Figure 3A,B). We also examined their effects in healthy mice. Blood‐chemistry analysis and HE staining of the heart, liver, spleen, lung, and kidney confirmed treatment safety. Even mice receiving a high‐dose administration of unloaded PBCA showed normal biochemical parameters and no evidence of tissue damage, implying that PBCA's potential toxicity is mitigated by metabolic clearance (Figure 3C,D).

To verify mNGF delivery to brain parenchyma, the protein was conjugated to FITC (green) and visualized by fluorescence microscopy. Pharmacokinetic studies indicate that mNGF reaches peak plasma concentration at 4.01 h, with a distribution half‐life (*kα*) of 1.87 h, an elimination half‐life (*kₑ*) of 4.71 h, and 87.3% absorption 24 h after intramuscular injection in rats. Accordingly, we harvested brains 4 h post‐TBI to test whether PBCA‐coated mNGF could cross the BBB under these conditions. As shown in Figure 3E, the “TBI + PBCA‐mNGF” group displayed many more green puncta in the cortex than the “Sham + PBCA‐mNGF” group, indicating efficient PBCA‐assisted transport into the CNS. Quantification (Figure 3I) further showed that free mNGF penetrated the parenchyma poorly; even with BBB disruption after TBI, only minimal fluorescence was detected.

To measure absolute delivery, we first established a standard curve for FITC‐labeled mNGF (Figure 3F):

$$Y=-105271.08714+2802011.08750\;X-91791.39446\;X^{2}$$

$$Y:\mathrm{OD}\;\text{values}\;X:\text{concentration}\;\left(\mathrm{\mu g}/\mathrm{mL}\right)\;R^{2}=0.99919$$

Using this curve, we converted cerebral OD readings to drug concentrations (Figure 3J). Parallel analyses—including a BMEC monolayer permeability test—confirmed that PBCA‐mNGF crossed the cellular barrier more readily than uncoated mNGF (Figure 3G,K). In vivo imaging further showed clear accumulation of PBCA‐mNGF around TBI lesions within the first 24 h after injection (Figure 3H,L). Together, these results demonstrate that PBCA encapsulation provides excellent biosafety and greatly enhances mNGF delivery to injured brain tissue.

### 3.3 PBCA‐mNGF Attenuated Glymphatic System Dysfunction 3 Days Following TBI

**FIGURE 4:**
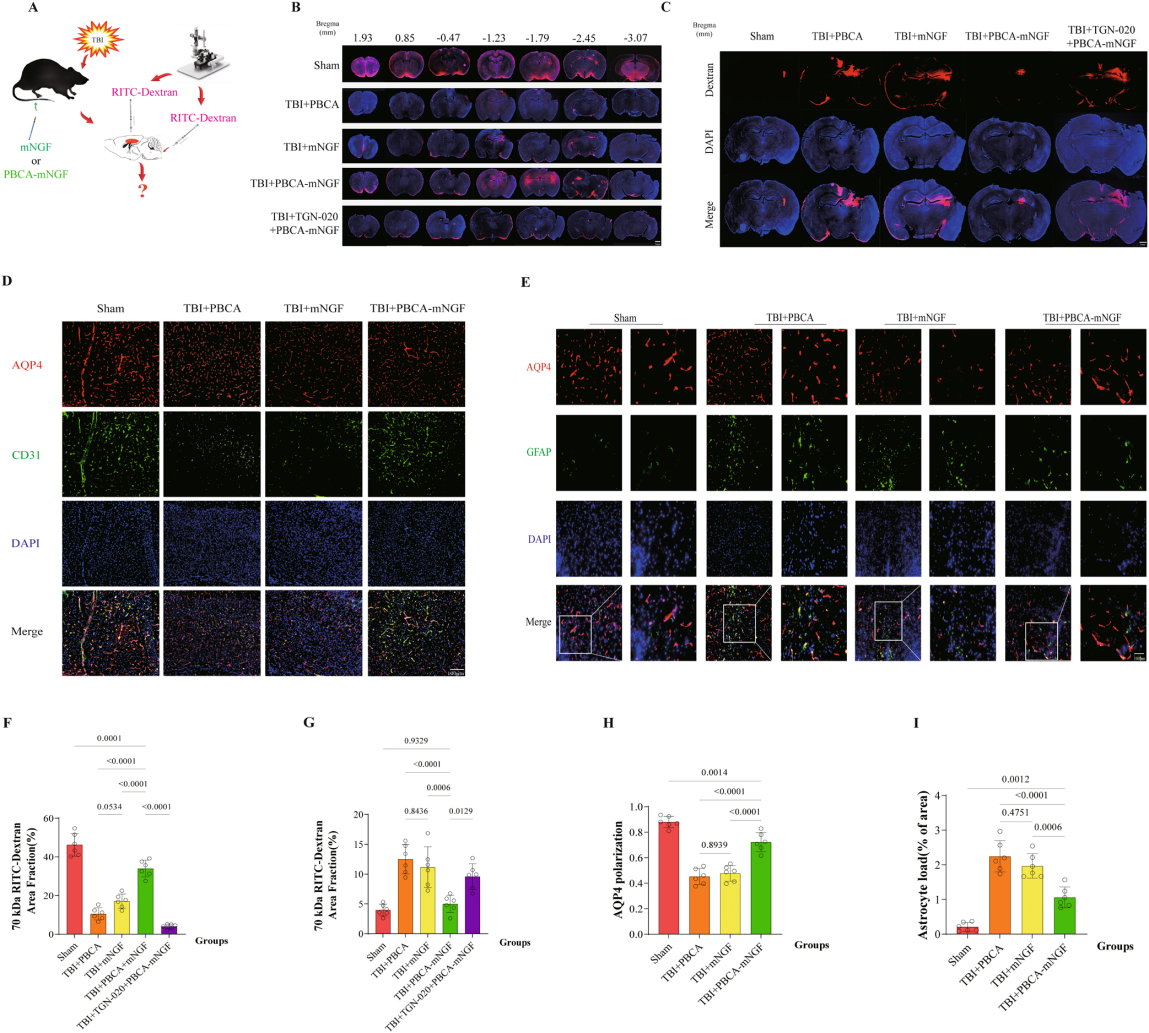
PBCA‐loaded mNGF attenuated glymphatic system dysfunction 3 days following TBI. (A) Schematic illustrating fluorescent‐tracer injection into the cisterna magna or brain parenchyma 3 days post‐TBI in the four experimental groups. (B) Representative coronal sections showing tracer influx: RITC‐dextran (red) and nuclei (DAPI, blue). Scale bar, 1 mm. (C) Representative sections showing tracer clearance under the same staining conditions. Scale bar, 1 mm. (D) Co‐immunofluorescence (co‐IF) for AQP4 (red) and CD31 (green) in peri‐lesional cortex; DAPI marks nuclei. Scale bar, 100 μm (*n* = 6 mice per group). (E) Co‐IF for AQP4 (red) and GFAP (green) in peri‐lesional cortex; regions of interest (ROIs) highlight AQP4‐astrocyte colocalization. Scale bars, 100 μm (200×) and 50 μm (400×) (*n* = 6). (F) Quantification of the RITC‐dextran influx area in −1.79 mm sections from panel B (*n* = 6). (G) Quantification of residual RITC‐dextran in −2.06 mm sections from panel C (*n* = 6). (H) Quantification of AQP4 polarization across groups (*n* = 6). (I) Quantification of GFAP‐positive area in whole‐brain sections (*n* = 6).

The exchange of cerebrospinal fluid (CSF) and interstitial fluid (ISF) is fundamental to glymphatic structure and function, supporting cerebral waste clearance [11]. Traumatic brain injury (TBI), however, disrupts this process. To evaluate the therapeutic impact of PBCA‐coated mNGF on CSF penetration along cerebral arteries, we slowly infused 10 μL of fluorescent tracer into the subarachnoid CSF at the cisterna magna of TBI mice. After a 30‐min circulation period, animals were euthanized and perfusion‐fixed. Fluorescence microscopy revealed markedly reduced tracer influx into parenchyma after TBI versus sham. Intramuscular mNGF modestly increased the fluorescent area, whereas PBCA‐mNGF produced a pronounced enhancement of perivascular tracer entry (Figure 4B).

To compare drug‐delivery modes for solute clearance, 0.5 μL of tracer was stereotactically injected into cortical parenchyma adjacent to the lesion. One hour later, residual fluorescence was quantified. Elevated tracer retention indicated impaired clearance. Post‐TBI brains contained significantly more residue; free mNGF produced minimal improvement, but PBCA‐mNGF markedly reduced tracer levels, signifying enhanced interstitial solute removal (Figure 4C).

AQP4 facilitates paravascular CSF recirculation and ISF clearance [38]. We assessed AQP4 polarization by co‐immunofluorescence (co‐IF) for AQP4 (red) and CD31 (green). Three days after TBI, perivascular AQP4 was greatly diminished. Free mNGF did not restore polarization, whereas PBCA‐mNGF substantially reinstated it (Figure 4D). We hypothesized that mNGF limits AQP4 depolarization, thereby improving glymphatic clearance. To test this, the AQP4 inhibitor TGN‐020 was administered to a TBI + PBCA‐mNGF subgroup. TGN‐020 disrupted CSF‐ISF exchange (Figure 4B,C), indicating that restored AQP4 polarization underlies mNGF's drainage benefit; the inhibitor abolished these gains (Figure 4F,G).

Reactive astrogliosis was evaluated by dual staining for GFAP (green) and AQP4 (red). GFAP expression rose sharply in the TBI + PBCA group, remained unchanged in the TBI + mNGF group, and decreased—though not to sham levels—in the PBCA‐mNGF group. High‐magnification images (white boxes) showed closer alignment of astrocytic end‐feet with AQP4 in Sham and TBI + PBCA‐mNGF cortices than in untreated or TBI + mNGF brains (Figure 4E). Taken together, these findings suggest that efficient mNGF delivery not only normalizes AQP4 distribution but may also alleviate downstream BBB damage.

### 3.4 PBCA‐mNGF Attenuated BBB Disruption 3 Days Following TBI

**FIGURE 5:**
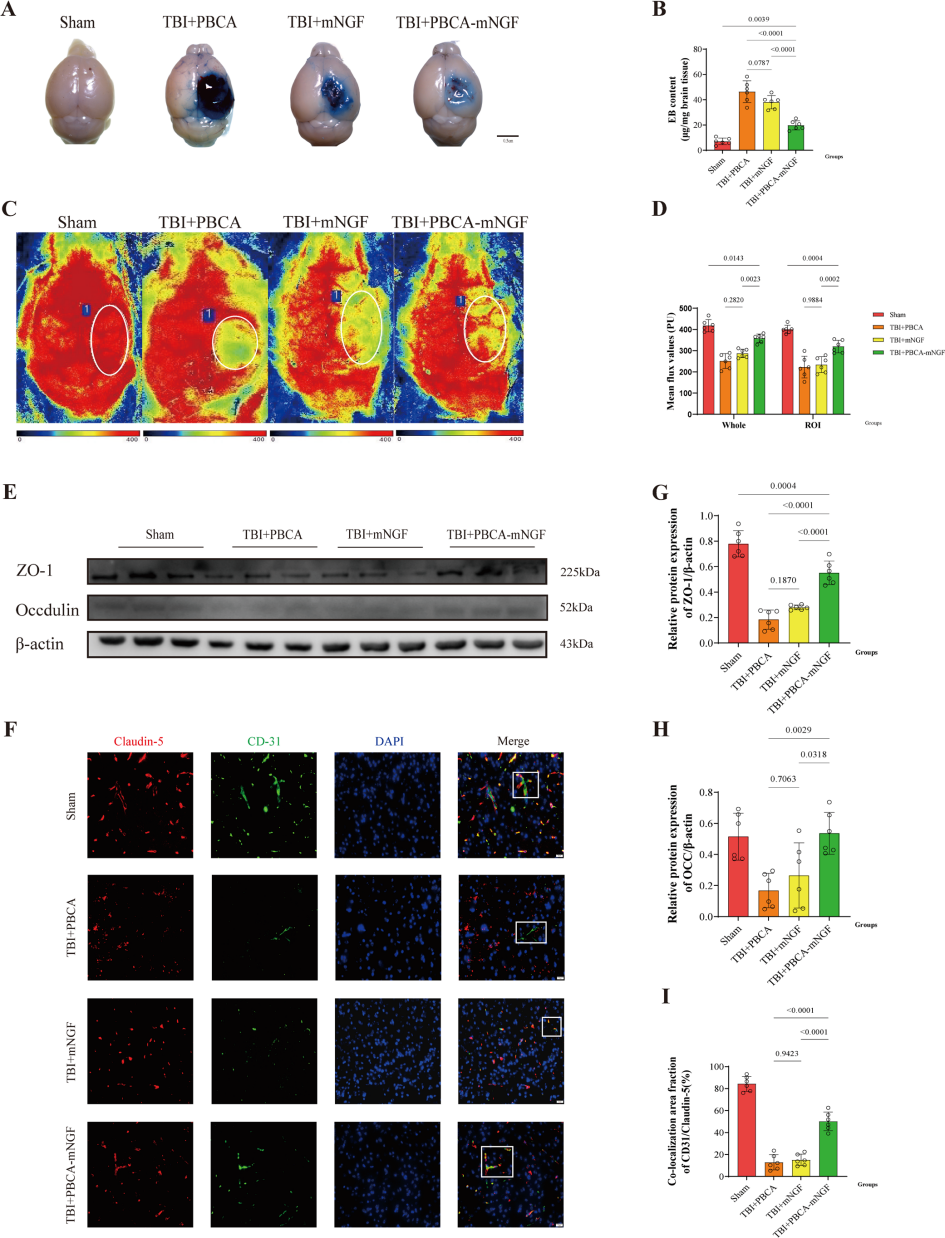
PBCA‐mNGF attenuated BBB disruption 3 days following TBI. (A) Representative images from the Evans blue (EB) extravasation assay. Scale bar, 0.5 cm. (B) Quantification of EB content in brain tissue (*n* = 6 mice per group). (C) Representative cerebral blood‐flow (CBF) images in sham and TBI mice. (D) Mean CBF flux values of the integral brain and ROI in sham and TBI cohorts. (E) Representative Western blots for ZO‐1 and occludin. (F) Co‐immunofluorescence for Claudin‐5 (red) and CD31 (green) in peri‐lesional cortex; DAPI marks nuclei. The region of interest (ROI) highlights Claudin‐5–vascular colocalization. Scale bar, 200 μm (*n* = 6). (G, H) Densitometric analysis of ZO‐1 and occludin, normalized to β‐actin. (I) Quantification of Claudin‐5/CD31 colocalized area in brain sections from the four groups (*n* = 6 mice per group).

We assessed BBB integrity—crucial for fluid exchange and solute clearance—using Evans blue (EB) extravasation. In the ipsilateral hemisphere, neither tissue reddening (erythrosis) nor EB leakage was appreciably reduced in the TBI + mNGF group compared with the TBI + PBCA group. By contrast, nanoparticle delivery markedly increased mNGF deposition in the parenchyma, mitigating TBI‐induced BBB injury. Consequently, the TBI + PBCA‐mNGF group exhibited less erythrosis, diminished EB extravasation, and lower EB content (Figure 5A,B).

Laser speckle imaging performed on day 3 post‐TBI revealed greater CBF signals in the PBCA‐mNGF group than in the conventional‐treatment group, although values remained below those of sham mice (Figure 5C,D). Western blotting showed that PBCA‐mNGF preserved tight‐junction proteins ZO‐1 and occludin, both essential for BBB integrity (Figure 5E,G,H). Co‐IF further demonstrated a larger Claudin‐5/CD31 overlap area in the TBI + PBCA‐mNGF group than in any other TBI group, corroborating improved barrier function (Figure 5F,I). Collectively, these findings indicate that PBCA carriers can curtail BBB disruption by enhancing mNGF delivery.

### 3.5 PBCA‐mNGF Played a Role in Neural Protection and Myelin Recovery After TBI

**FIGURE 6:**
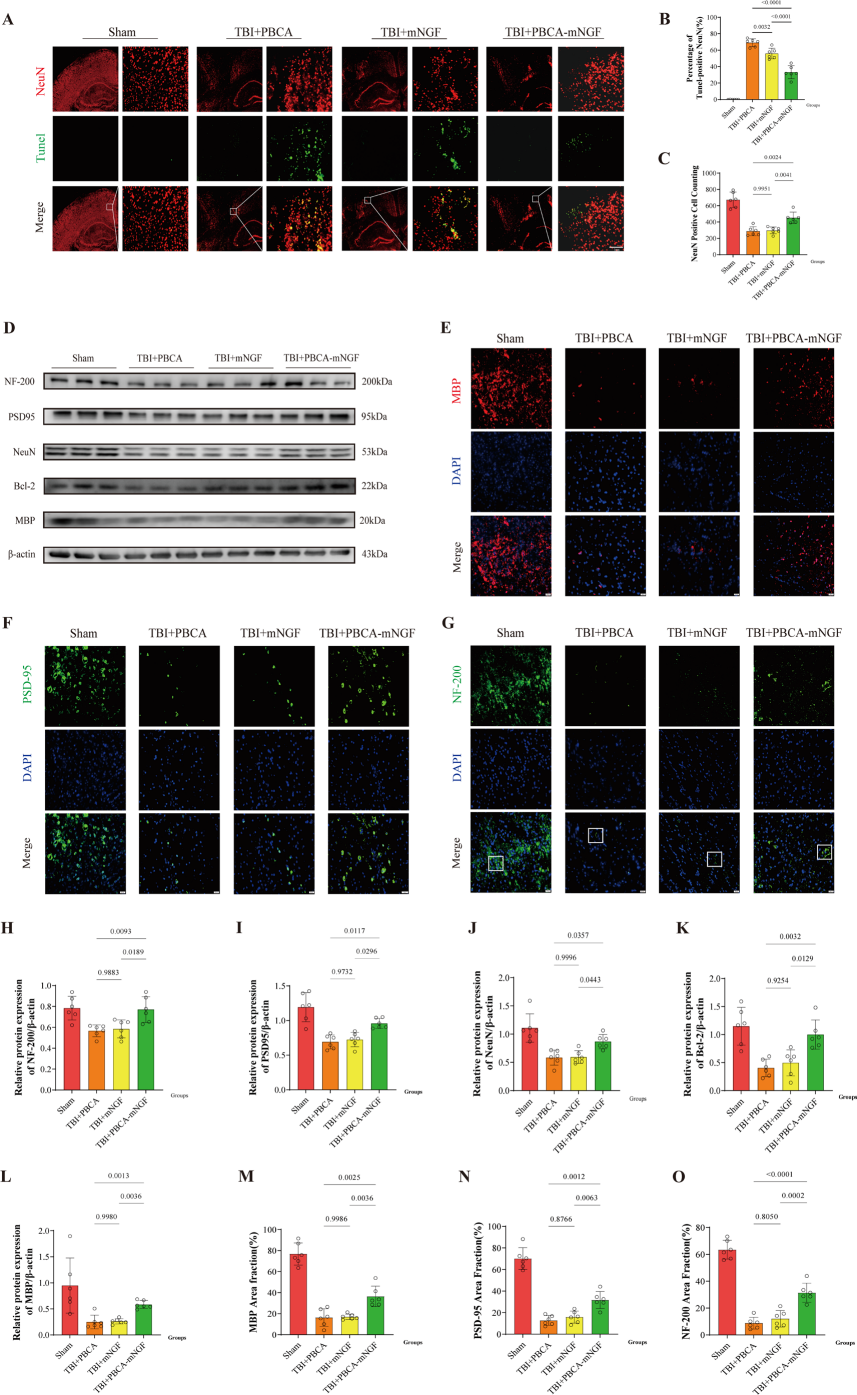
With the assistance of PBCA, mNGF more effectively inhibited neuronal death and promoted the expression of myelin‐related proteins 3 days post‐TBI. (A) TUNEL (green) and NeuN (red) co‐staining in injured cortex at 40× and 400× magnification; white boxes mark regions of interest (ROIs). TUNEL^+^/NeuN^+^ cells denote apoptotic neurons. Scale bar, 1 mm. (B) Percentage of apoptotic neurons in each ROI (*n* = 6 mice per group). (C) Counts of NeuN‐positive neurons per ROI (*n* = 6). (D) Representative Western blots for NeuN, MBP, PSD‐95, NF‐200, and Bcl‐2; β‐actin serves as the loading control. (E) Immunofluorescence (IF) for MBP (red) around the lesion; DAPI marks nuclei. Scale bar, 200 μm (*n* = 6). (F) IF for PSD‐95 (green) in the same region; DAPI counterstain. Scale bar, 200 μm (*n* = 6). (G) IF for NF‐200 (green) near the lesion; ROI highlights fibers with greater integrity. Scale bar, 200 μm (*n* = 6). (H–L) Densitometric quantification of NF‐200 (H), PSD‐95 (I), NeuN (J), Bcl‐2(K), and MBP(L), normalized to β‐actin (*n* = 6). (M–O) Quantification of MBP (M), PSD‐95 (N), and NF‐200 (O) immunoreactive areas across groups (*n* = 6 mice per group).

TUNEL staining showed that conventional mNGF administration minimally reduced neuronal death, whereas the TBI + PBCA‐mNGF group displayed fewer apoptotic neurons, though counts remained above sham levels (Figure 6A,C,J). Western blot analyses revealed lower NF‐200 and Bcl‐2 expression in the TBI + mNGF group than in the TBI + PBCA controls; averaging across groups confirmed that PBCA‐mNGF more effectively supported neuronal survival and regeneration (Figure 6D).

PBCA‐mNGF also enhanced myelin‐related proteins. MBP—a principal CNS myelin component—was up‐regulated only in the PBCA‐mNGF group, whereas free mNGF produced no significant change. Postsynaptic PSD‐95 and axonal NF‐200 likewise increased more robustly with PBCA‐mNGF. Immunofluorescence corroborated these findings: fluorescence signals for MBP, PSD‐95, and NF‐200 were highest in the PBCA‐mNGF cohort (Figure 6E–G,M–O). Notably, NF‐200 fibers appeared thicker and more intact in sham brains, fragmented after TBI, and partially restored after PBCA‐mNGF treatment—suggesting that an adequate dose of mNGF can facilitate axonal and myelin repair. Conversely, certain proteins such as MBP still trailed sham levels, implying that full recovery may require additional time for neurogenesis and axogenesis.

Preliminary data (Figure S1A,B) indicate that mNGF also dampens microglial activation, an effect amplified by nanoparticle delivery; however, the precise neuroprotective mechanism remains to be elucidated.

### 3.6 “TBI + PBCA‐mNGF” Group Got Better Recovery of Motor and Cognitive Function Post‐TBI

**FIGURE 7:**
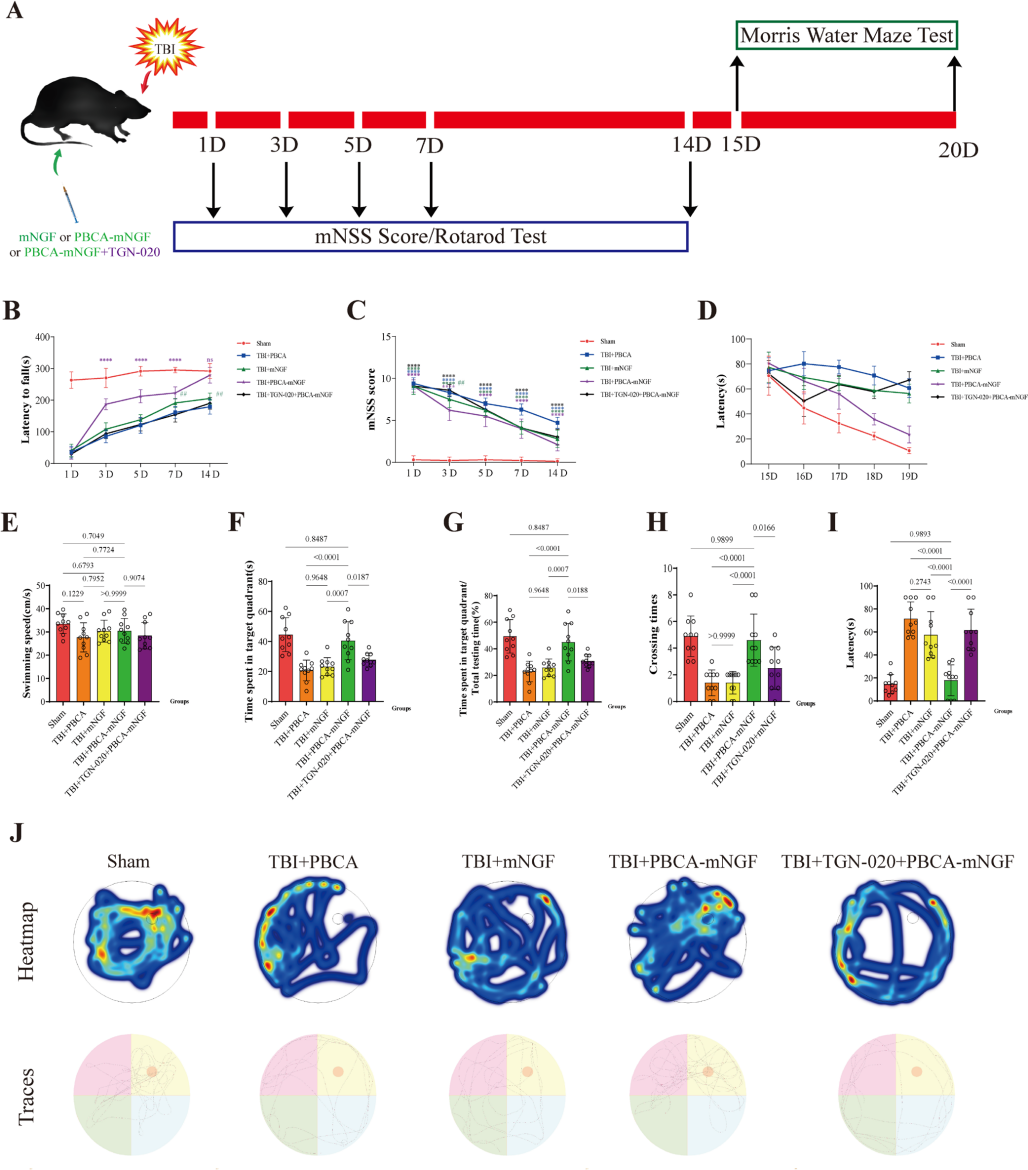
“TBI + PBCA‐mNGF” group demonstrated improved recovery of motor and cognitive function post‐TBI. (A) Timeline of behavioral testing. Modified Neurological Severity Scores (mNSS) and rotarod assessments were performed on days 1, 3, 5, 7, and 14; Morris water‐maze (MWM) trials began on day 15 and ended on day 20. (B) Rotarod performance (latency to fall) for each group (*n* = 10 mice per group). (C) mNSS across the same time points (*n* = 10). (D) Latency to locate the hidden platform during MWM training days 15–19 (*n* = 10). (E–I) Additional MWM outcomes—time in the target quadrant, platform crossings, path efficiency, dwell time, and swim speed—evaluating spatial learning and memory (*n* = 10). (J) Representative heatmaps and swim‐path trajectories illustrating search patterns for each group (*n* = 10).

To determine how mNGF influences post‐TBI recovery, we performed a battery of motor and cognitive assays (Figure 7A). In the rotarod test, the TBI + PBCA‐mNGF group improved markedly within 3 days, outperforming the other TBI groups, although performance still lagged behind the Sham group. As recovery progressed, the gap between PBCA‐treated and Sham mice narrowed; by day 20, motor capacity no longer differed significantly between these two groups. Conventional mNGF administration modestly enhanced performance versus untreated TBI mice but did not reach statistical significance at any time point (Figure 7B).

Consistent with these findings, mNSS differed significantly between the two mNGF groups on day 3; thereafter, scores in all TBI cohorts trended downward without further significant separation (Figure 7C). For long‐term cognition, we used the MWM. Outcome variables—swim speed, time in the target quadrant, platform crossings, and latency—were analyzed to control for motor confounds. PBCA‐mNGF mice performed comparably to Sham animals and markedly better than the other TBI groups, whereas free mNGF produced only minor gains (Figure 7D–I). Heatmaps and path traces confirm that PBCA‐treated mice pursued direct routes and spent more time in the target quadrant, indicating restored spatial memory (Figure 7J).

Finally, co‐administration of the AQP4 inhibitor TGN‐020 negated the benefits of PBCA‐mNGF: drainage dysfunction and metabolite accumulation re‐emerged, and behavioral as well as cognitive scores deteriorated (Figure 7B–J). These results underscore that PBCA‐mediated mNGF delivery effectively mitigates TBI‐related motor and cognitive deficits, whereas AQP4 inhibition reverses these gains.

## 4 Discussion

Few studies have examined mNGF therapy in TBI mice, largely because large molecules are difficult to deliver to the cerebral cortex. Our data show that delivery efficiency in the TBI + PBCA‐mNGF group exceeded that of all other groups. Fluorescence imaging (Figure 3E) further revealed no significant difference between the TBI + mNGF and Sham + PBCA‐mNGF groups, implying that BBB disruption alone permits only limited, transient entry of large molecules into brain parenchyma, whereas PBCA markedly enhances delivery to achieve therapeutic concentrations. Consequently, our work is among the few demonstrations that mNGF can mitigate CNS injury. Post‐TBI, glymphatic inflow and outflow are severely compromised, causing neurotoxic substrates and TBI‐specific markers to accumulate in blood [39, 40]. Our finding that mNGF improves both processes is therefore novel. Reactive astrogliosis and loss of perivascular AQP4 polarization are central to this dysfunction. Astrogliosis is heterogeneous, featuring molecular, cellular, and functional shifts and often accompanied by intermediate‐filament up‐regulation and astrocyte hypertrophy [41]. Changes in AQP4 polarization—particularly 14–28 days after injury—correlate closely with astrogliosis severity [42]. AQP4 function depends on its polarized distribution, which in turn relies on an intact dystroglycan complex (DG) at astrocytic end‐feet [43]. TBI‐induced astrogliosis disrupts astrocyte morphology and DG integrity, causing AQP4 depolarization and misalignment with the basement membrane. Although these associations are well documented, additional evidence is needed to clarify the precise causal links between astrogliosis and AQP4 depolarization.

Moreover, our study is the first to demonstrate that mNGF acts as a neuroprotective agent by preventing AQP4 depolarization and limiting reactive astrocyte proliferation. We also observed that mNGF up‐regulates genes supporting BBB structure while down‐regulating those associated with neuronal apoptosis. Three days after TBI, the PBCA‐mNGF group exhibited the best recovery of CBF and the least EB leakage; the other TBI groups still showed marked ischemia and substantial dye extravasation. In parallel, tight‐junction proteins remained better preserved in PBCA‐mNGF brains, suggesting maintained BBB integrity. Although mNGF is well known to promote peripheral axon regeneration, its CNS role is less understood. Because PBCA nanocarriers traverse the BBB, we hypothesized that mNGF could mitigate acute CNS injury. Indeed, PBCA‐mNGF reduced neuronal loss and apoptosis‐related protein levels, implying protection against neurodegeneration. This effect may involve microglial deactivation: CD68/Iba‐1 co‐staining revealed post‐TBI microglial swelling that was attenuated by mNGF. Clarifying this mechanism will be a priority in future work. Importantly, PBCA‐mNGF also improved cognitive performance and motor recovery—outcomes that could translate into better patient quality of life and social reintegration [38]. These benefits likely stem from enhanced glymphatic drainage, improved perfusion of injured tissue, and reduced neuronal apoptosis [25, 44]. To probe the mechanism, we administered the AQP4 inhibitor TGN‐020; prognosis worsened, supporting a pivotal role for AQP4 polarization in glymphatic function and waste clearance after TBI. How mNGF preserves AQP4 polarity remains unclear. Astrocyte end‐foot swelling is tied to aberrant calcium signaling [45], and mNGF can stabilize neuronal calcium homeostasis [46]; thus, mNGF might also limit astrocytic swelling and maintain AQP4 alignment—a hypothesis that warrants testing. Although clinical data on mNGF for TBI remain limited, our findings show that adequate parenchymal delivery can ameliorate injury in a mouse model. The neuroprotective effect correlates with brain mNGF dose, underscoring the importance of efficient delivery; nanocrystallization appears to be a promising strategy.

Retrospective studies have emphasized restoring the cerebral glymphatic system to correct structural and functional deficits, and numerous interventions have been reported to improve TBI prognosis. For example, exogenous interleukin‐33 (IL‐33) promotes metabolic‐waste clearance by enhancing drainage from meningeal lymphatics to deep cervical lymph nodes [47]. Likewise, cannabidiol from *Cannabis sativa* mitigates cognitive dysfunction by inhibiting AQP4 depolarization and thereby facilitating CSF–ISF exchange [48]. Activation of specific receptors—such as the cerebral glucagon‐like peptide‐1 receptor—also modulates glymphatic flow and reduces post‐TBI complications [5]. Beyond pharmacologic agents, engineered nanoenvironments have been shown to remodel the glymphatic pathway and improve long‐term outcomes in TBI mice [49]. Collectively, these findings underscore the glymphatic system as a prime therapeutic target. Although nerve growth factor is a well‐studied neuroprotective molecule, its utility in glymphatic modulation has been limited by its high molecular weight, which restricts BBB passage and, consequently, its anti‐apoptotic and axon‐promoting functions. Effective CNS delivery is therefore the first hurdle. In our study, we designed a lipid‐based nanocarrier to ferry FITC‐labeled mNGF across the BBB and confirmed its neuroprotective activity. Both the PBCA carrier and the FITC label are well‐validated biomedical tools for tracing macromolecular drugs in target tissues. Prior work has shown that PBCA can convey large proteins such as horseradish peroxidase into the brain parenchyma [12], guiding our strategy to deliver mNGF to the cortex. However, the high hydrophilicity of mNGF contributed to a low drug‐loading capacity, leaving many PBCA particles unloaded. Earlier studies reported that PBCA payload increases with drug hydrophobicity [50]. Unloaded PBCA particles also raise translational concerns. Although PBCA is considered safe in rodents, its human safety profile remains uncertain—for instance, whether excessive PBCA might induce hyperlipidemia. These issues must be resolved before clinical use. Nonetheless, our work provides a viable approach for overcoming BBB restrictions, and our data corroborate the neuroprotective effect of mNGF delivered via PBCA carriers.

Nanocrystallization is not the only way to enhance NGF penetration. Under hypoxic–ischemic conditions, β‐NGF—a synthetic NGF analog—distributes more readily in juvenile than in adult rats, implying that delivery efficiency depends on BBB integrity [51]. Electronic acupuncture has likewise been shown to raise endogenous NGF levels and permit its passage across the BBB in middle cerebral artery–occluded rats [52]. Thus, NGF can traverse the BBB in certain pathological states, but its delivery under normal physiological conditions remains uncertain. Intranasal administration is another viable route: previous work delivered NGF intranasally to the pediatric CNS [44]. Comparing intranasal and intramuscular delivery efficiencies will be a focus of future research. In adults with severe nasal or facial trauma—or with nasotracheal tubes and gastric catheters—nasal delivery may be impractical, making nanoparticles especially attractive. Owing to their mucoadhesive and penetrating properties, PBCA nanoparticles have even been explored for oral insulin delivery [53]. Here, we confirmed that mNGF reaches the adult mouse cortex via PBCA carriers, suggesting broad applicability. Evidence also indicates that PBCA is a reliable, convenient, and practical drug carrier with strong clinical potential. Our study has limitations. First, we examined only the acute phase of TBI; because neuronal and axonal regeneration takes time, future work should determine whether extended observation narrows the performance gap between conventional and PBCA‐mNGF delivery. Second, the precise route by which PBCA‐mNGF accesses the glymphatic system requires clarification. Finally, delivery parameters must be optimized for both DL and EE, rather than favoring one at the expense of the other.

## 5 Conclusion

This study demonstrates that nanoparticle carriers markedly enhance the delivery of mNGF to brain parenchyma, thereby conferring neuroprotection. In the acute phase of TBI, mNGF appears to restore glymphatic function, preserve BBB integrity, and reduce injury‐induced apoptosis—all factors that contribute to improved prognosis in TBI mice. Accordingly, nanoparticle‐mediated mNGF delivery warrants further investigation as a promising therapeutic strategy for traumatic brain injury.

## Ethics Statement

The ethics approval statement was labeled as No. IRB2025‐DW‐41. All the animal experimental procedures described in this study were approved by the Animal Care and Use Committee of Tianjin Medical University General Hospital, Tianjin, China.

## Conflicts of Interest

The authors declare no conflicts of interest.

## Supporting information


**Figure S1:** mNGF inhibited the activation of microglial 3 days following TBI. (A) Co‐immunofluorescence for Iba‐1 (red) and CD68 (green) in the peri‐lesional cortex; DAPI marks nuclei. Scale bar, 200 μm (*n* = 6). (B) Quantification of activated microglial area fraction in brain sections from the four groups (*n* = 6).

## Data Availability

The data that support the findings of this study are available from the corresponding author upon reasonable request.
