# Supplementary material for: A Drug Delivery to Improve Prognosis of Traumatic Brain Injury Mice Through Mouse‐Derived Nerve Growth Factor Coated by a Nanoparticle
Source: CNS Neurosci Ther. 2025 Oct 2;31(10):e70603. doi: 10.1111/cns.70603 (PMC12491777; doi:10.1111/cns.70603)
Supplement: Supplementary file 1 — Figure S1: mNGF inhibited the activation of microglial 3 days following TBI. (A) Co‐immunofluorescence for Iba‐1 (red) and CD68 (green) in the peri‐lesional cortex; DAPI marks nuclei. Scale bar, 200 μm (n = 6). (B) Quantification of activated microglial area fraction in brain sections from the four groups (n = 6). [file CNS-31-e70603-s001.docx]

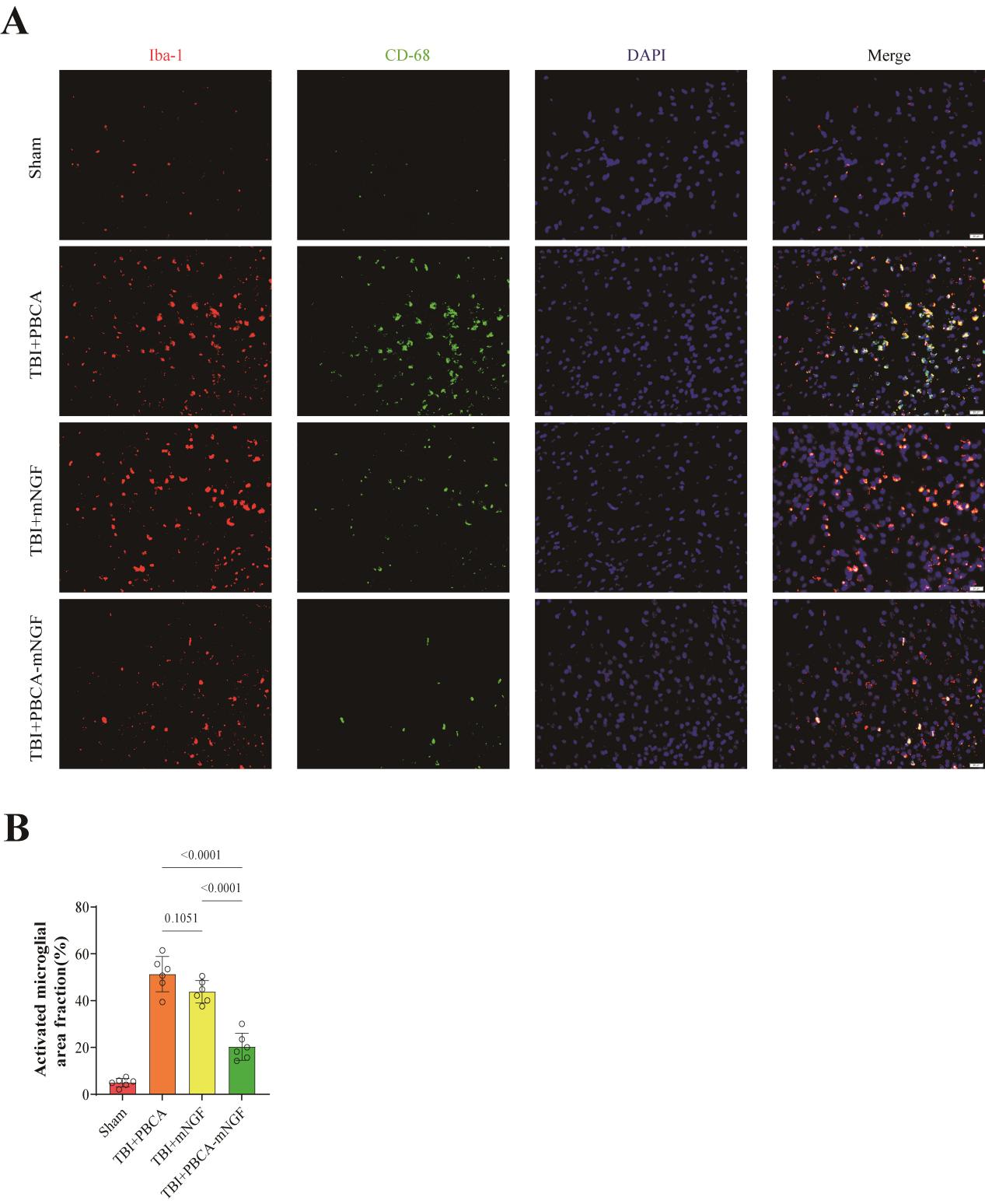


**Figure S mNGF inhibited the activation of microglial 3 days following TBI.**

(A)Co‑immunofluorescence for Iba-1 (red) and CD68 (green) in peri‑lesional cortex; DAPI marks nuclei. Scale bar, 200 µm (n = 6).

(B)Quantification of activated microglial area fraction in brain sections from the four groups (n = 6 ).
